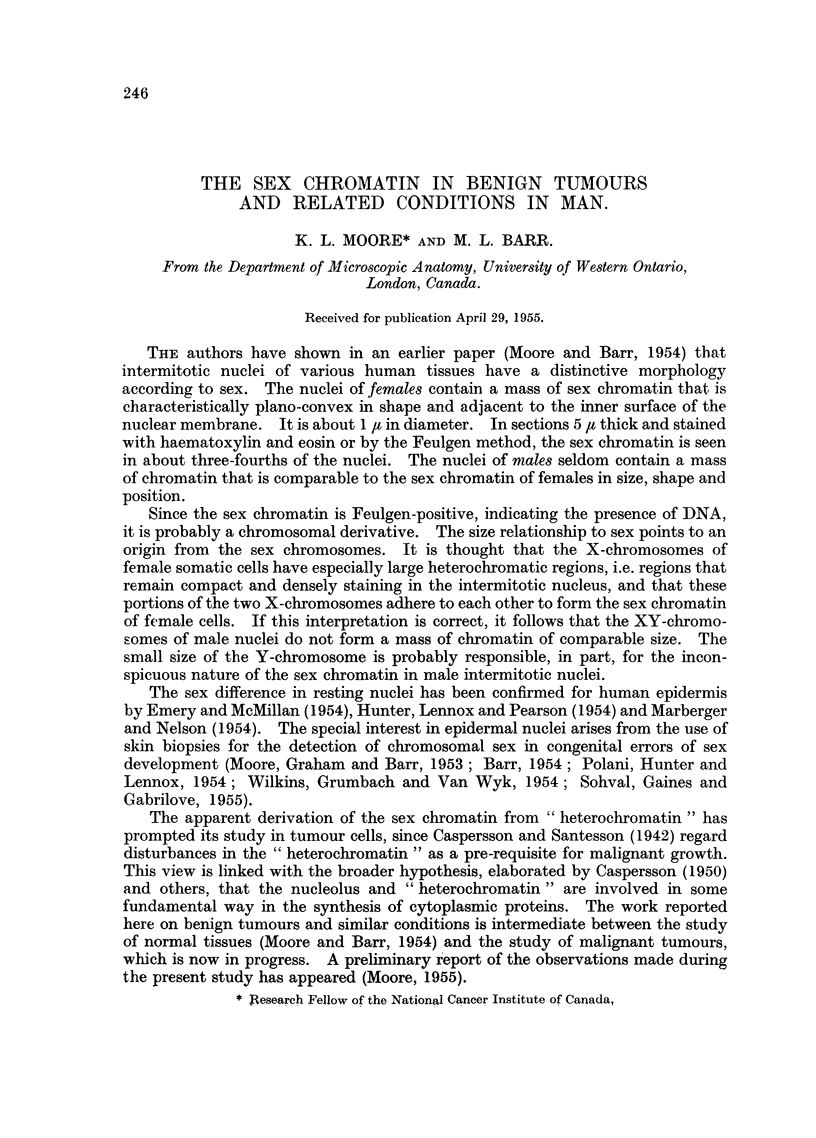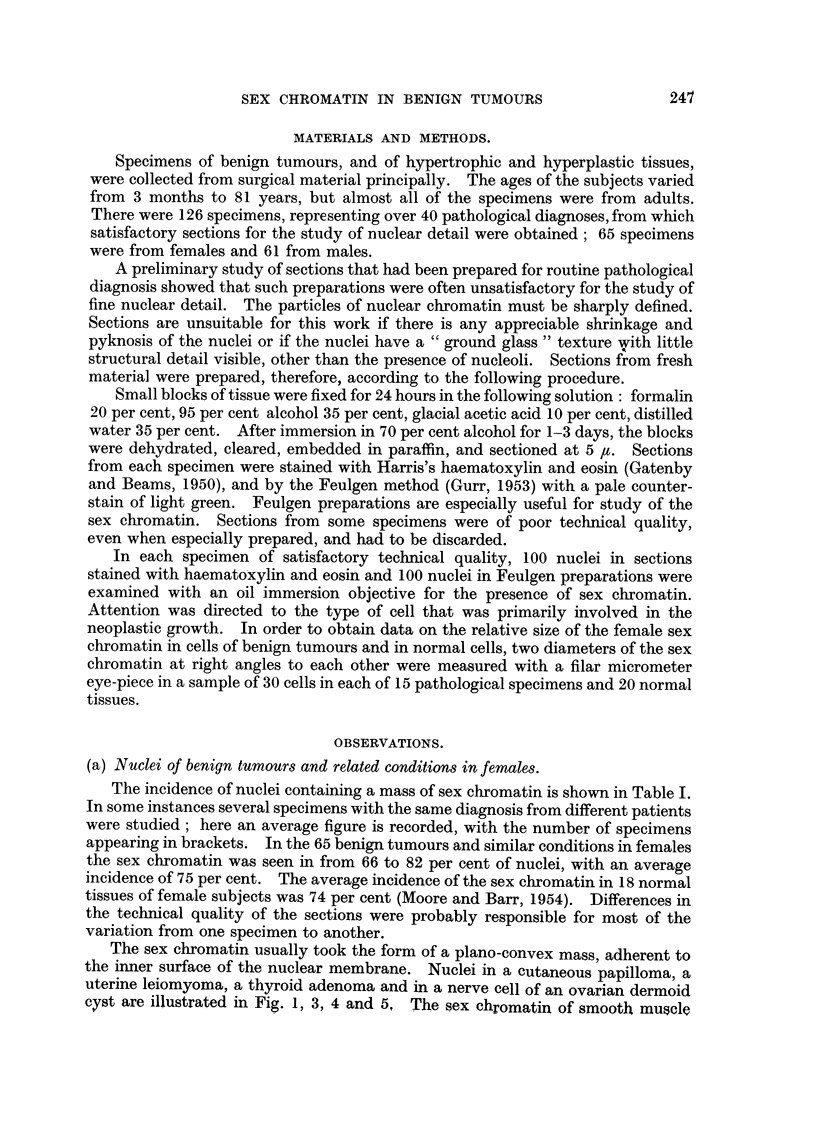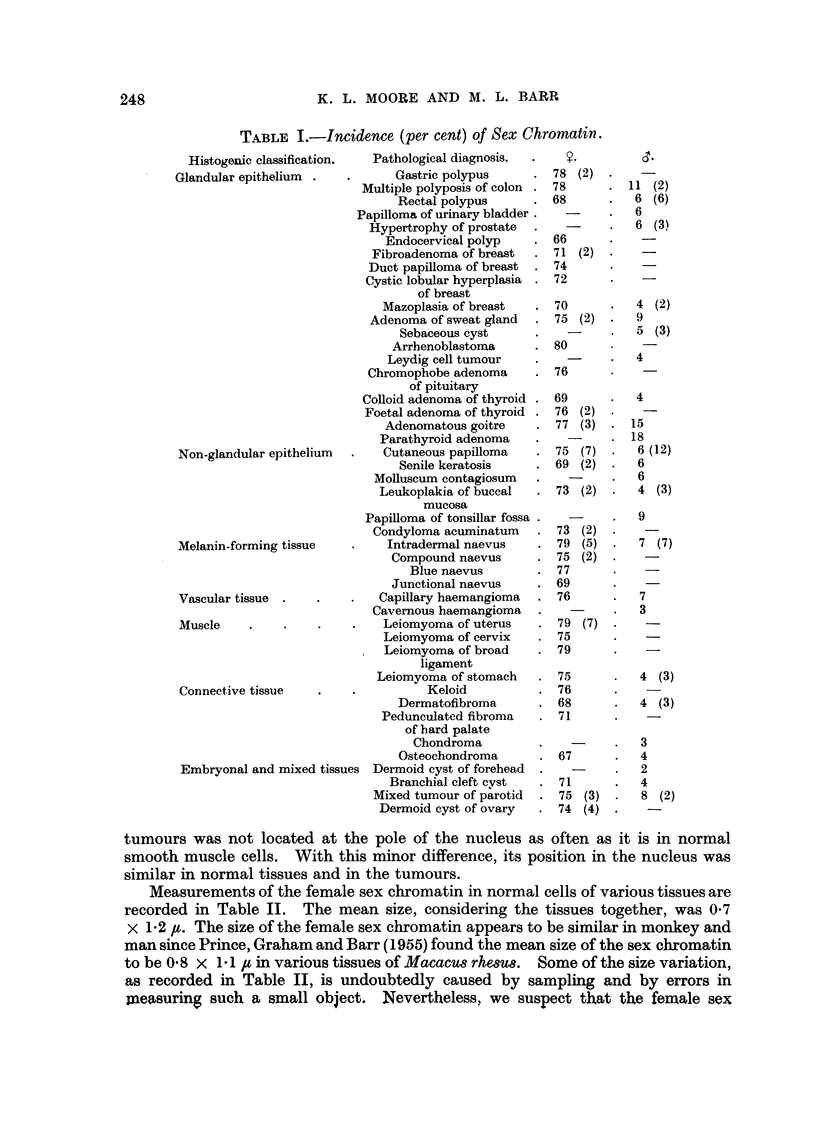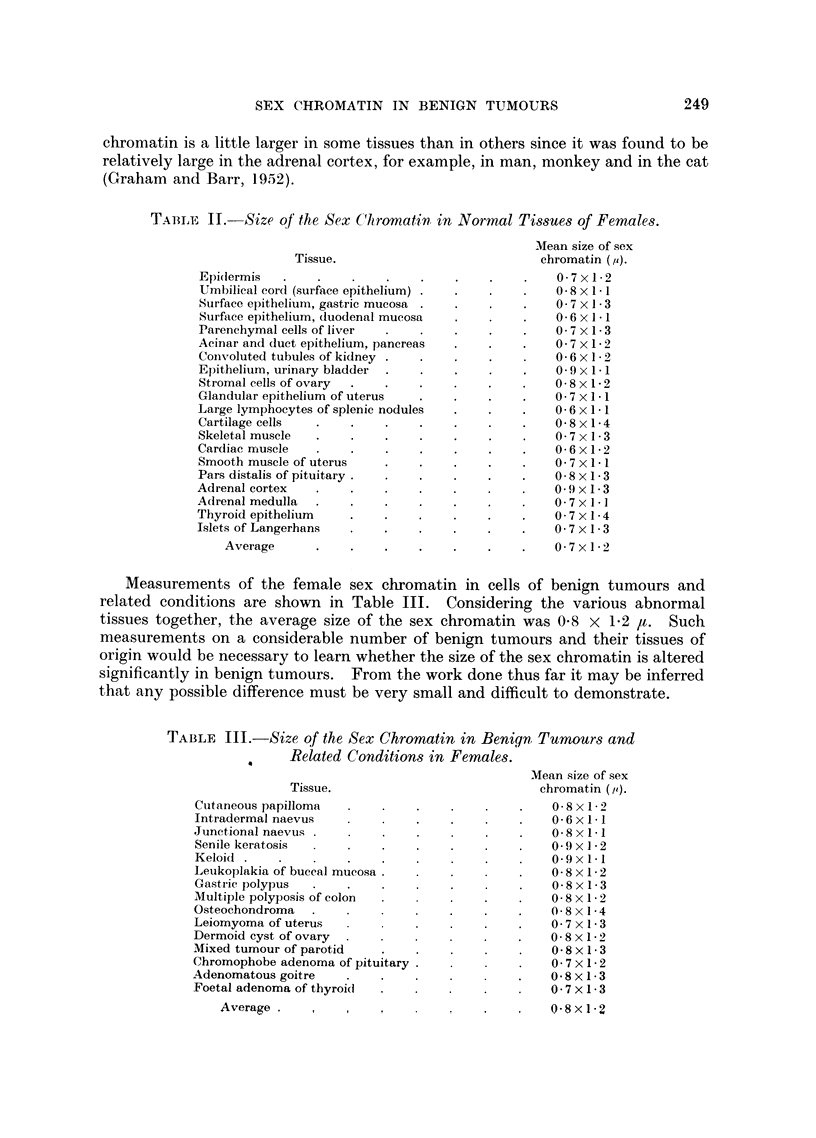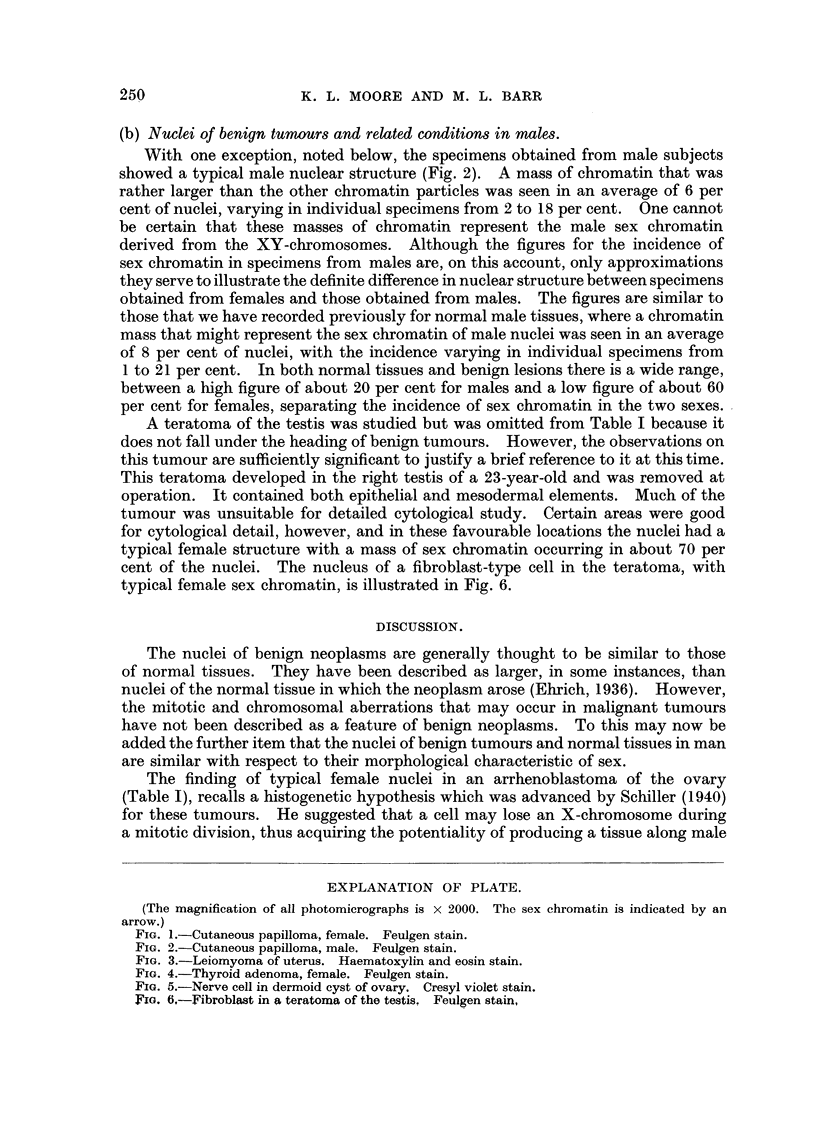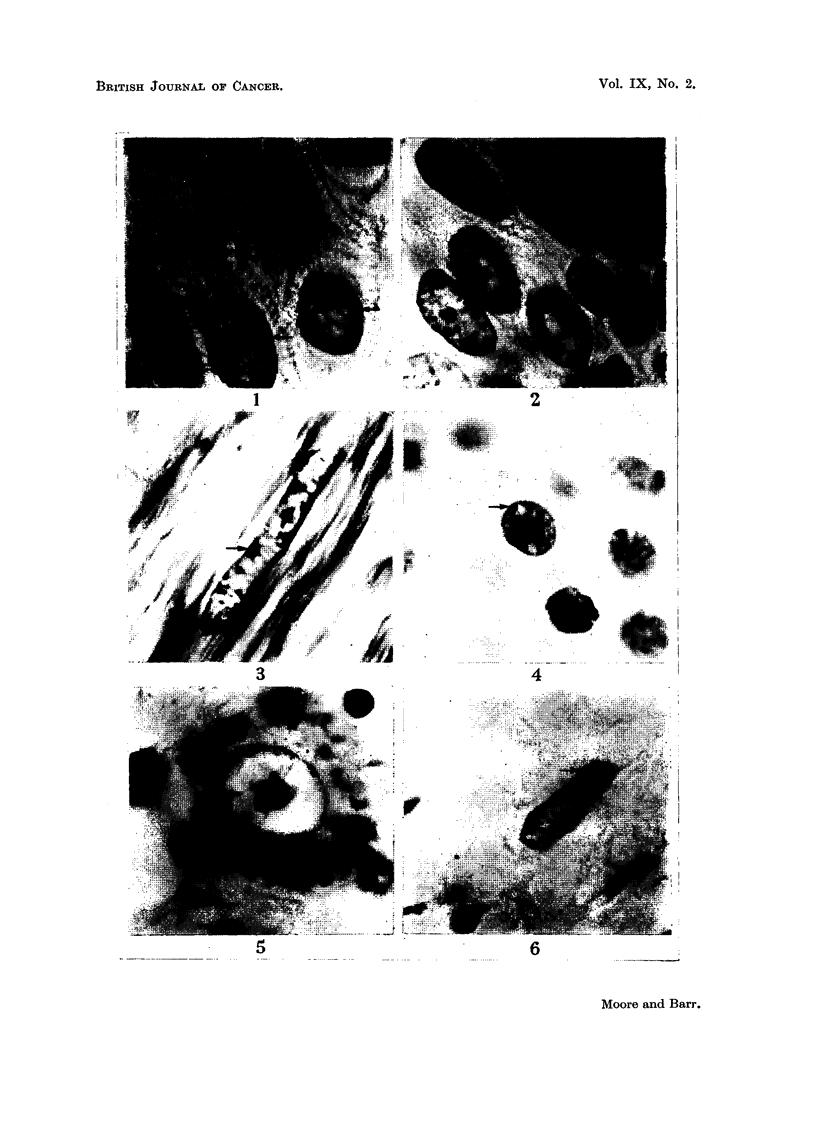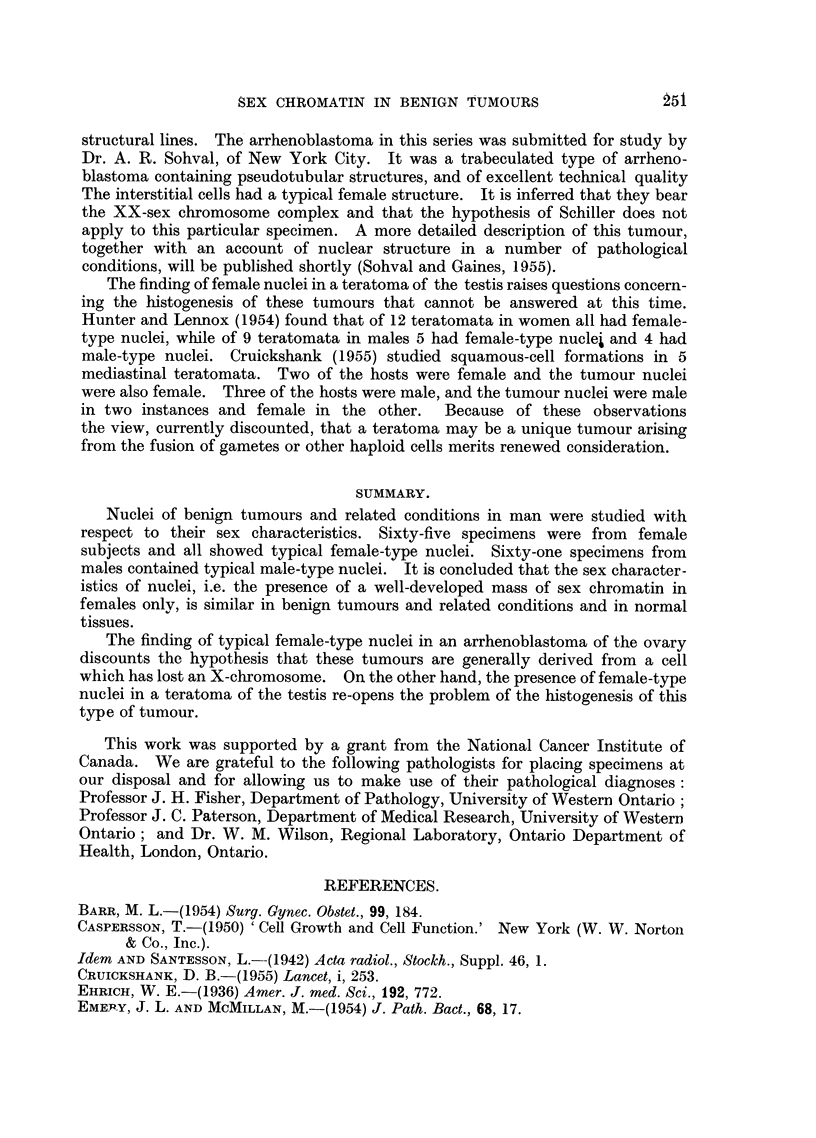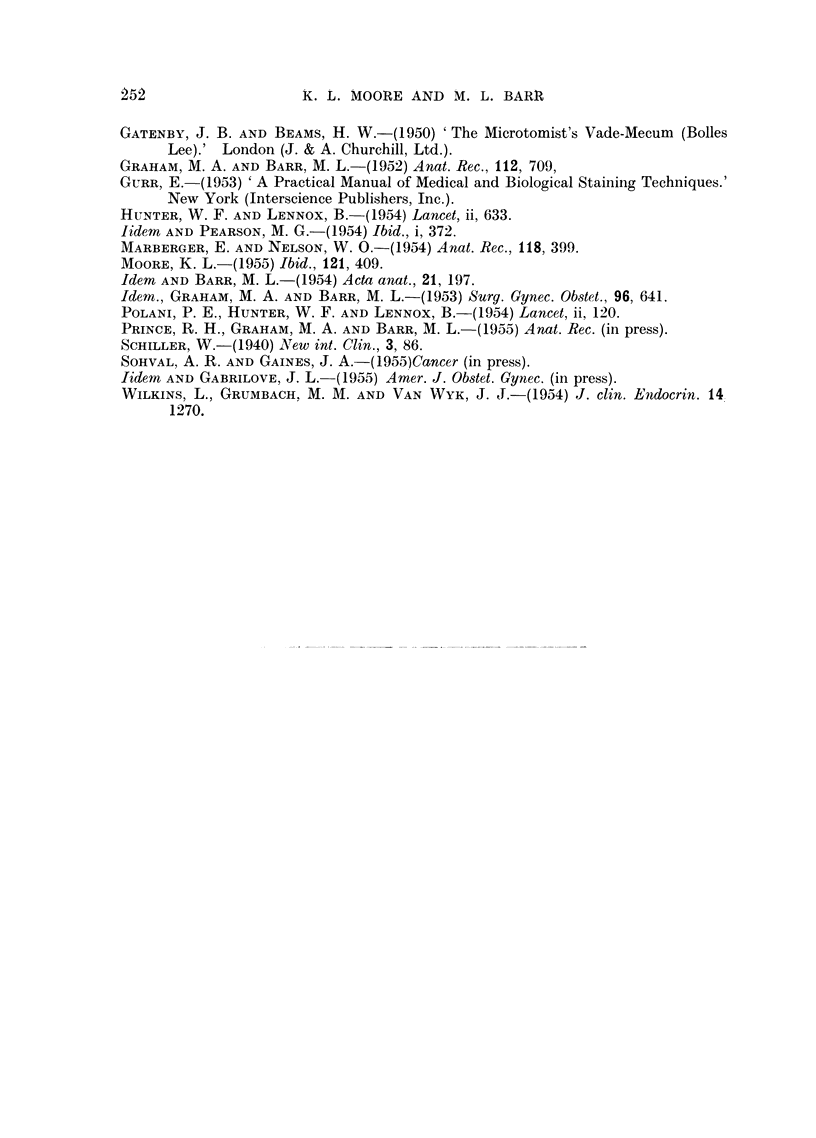# The Sex Chromatin in Benign Tumours and Related Conditions in Man

**DOI:** 10.1038/bjc.1955.21

**Published:** 1955-06

**Authors:** K. L. Moore, M. L. Barr

## Abstract

**Images:**


### THE SEX CHROMATIN IN BENIGN TUMOURS AND RELATED CONDITIONS IN MAN.

K. L. MOORE* AND M. L. BARR.

*From the Department of Microscopic Anatomy, University of Western Ontario, London, Canada.*

Received for publication April 29, 1955.

THE authors have shown in an earlier paper (Moore and Barr, 1954) that intermitotic nuclei of various human tissues have a distinctive morphology according to sex. The nuclei of *females* contain a mass of sex chromatin that is characteristically plano-convex in shape and adjacent to the inner surface of the nuclear membrane. It is about 1 $\mu$ in diameter. In sections 5 $\mu$ thick and stained with haematoxylin and eosin or by the Feulgen method, the sex chromatin is seen in about three-fourths of the nuclei. The nuclei of *males* seldom contain a mass of chromatin that is comparable to the sex chromatin of females in size, shape and position.

Since the sex chromatin is Feulgen-positive, indicating the presence of DNA, it is probably a chromosomal derivative. The size relationship to sex points to an origin from the sex chromosomes. It is thought that the X-chromosomes of female somatic cells have especially large heterochromatic regions, i.e. regions that remain compact and densely staining in the intermitotic nucleus, and that these portions of the two X-chromosomes adhere to each other to form the sex chromatin of female cells. If this interpretation is correct, it follows that the XY-chromosomes of male nuclei do not form a mass of chromatin of comparable size. The small size of the Y-chromosome is probably responsible, in part, for the inconspicuous nature of the sex chromatin in male intermitotic nuclei.

The sex difference in resting nuclei has been confirmed for human epidermis by Emery and McMillan (1954), Hunter, Lennox and Pearson (1954) and Marberger and Nelson (1954). The special interest in epidermal nuclei arises from the use of skin biopsies for the detection of chromosomal sex in congenital errors of sex development (Moore, Graham and Barr, 1953 ; Barr, 1954 ; Polani, Hunter and Lennox, 1954 ; Wilkins, Grumbach and Van Wyk, 1954 ; Sohval, Gaines and Gabrilove, 1955).

The apparent derivation of the sex chromatin from " heterochromatin " has prompted its study in tumour cells, since Caspersson and Santesson (1942) regard disturbances in the " heterochromatin " as a pre-requisite for malignant growth. This view is linked with the broader hypothesis, elaborated by Caspersson (1950) and others, that the nucleolus and " heterochromatin " are involved in some fundamental way in the synthesis of cytoplasmic proteins. The work reported here on benign tumours and similar conditions is intermediate between the study of normal tissues (Moore and Barr, 1954) and the study of malignant tumours, which is now in progress. A preliminary report of the observations made during the present study has appeared (Moore, 1955).

* Research Fellow of the National Cancer Institute of Canada.

### MATERIALS AND METHODS.

Specimens of benign tumours, and of hypertrophic and hyperplastic tissues, were collected from surgical material principally. The ages of the subjects varied from 3 months to 81 years, but almost all of the specimens were from adults. There were 126 specimens, representing over 40 pathological diagnoses, from which satisfactory sections for the study of nuclear detail were obtained ; 65 specimens were from females and 61 from males.

A preliminary study of sections that had been prepared for routine pathological diagnosis showed that such preparations were often unsatisfactory for the study of fine nuclear detail. The particles of nuclear chromatin must be sharply defined. Sections are unsuitable for this work if there is any appreciable shrinkage and pyknosis of the nuclei or if the nuclei have a " ground glass " texture with little structural detail visible, other than the presence of nucleoli. Sections from fresh material were prepared, therefore, according to the following procedure.

Small blocks of tissue were fixed for 24 hours in the following solution : formalin 20 per cent, 95 per cent alcohol 35 per cent, glacial acetic acid 10 per cent, distilled water 35 per cent. After immersion in 70 per cent alcohol for 1–3 days, the blocks were dehydrated, cleared, embedded in paraffin, and sectioned at 5 $\mu$. Sections from each specimen were stained with Harris's haematoxylin and eosin (Gatenby and Beams, 1950), and by the Feulgen method (Gurr, 1953) with a pale counter-stain of light green. Feulgen preparations are especially useful for study of the sex chromatin. Sections from some specimens were of poor technical quality, even when especially prepared, and had to be discarded.

In each specimen of satisfactory technical quality, 100 nuclei in sections stained with haematoxylin and eosin and 100 nuclei in Feulgen preparations were examined with an oil immersion objective for the presence of sex chromatin. Attention was directed to the type of cell that was primarily involved in the neoplastic growth. In order to obtain data on the relative size of the female sex chromatin in cells of benign tumours and in normal cells, two diameters of the sex chromatin at right angles to each other were measured with a filar micrometer eye-piece in a sample of 30 cells in each of 15 pathological specimens and 20 normal tissues.

### OBSERVATIONS.

### (a) *Nuclei of benign tumours and related conditions in females.*

The incidence of nuclei containing a mass of sex chromatin is shown in Table I. In some instances several specimens with the same diagnosis from different patients were studied ; here an average figure is recorded, with the number of specimens appearing in brackets. In the 65 benign tumours and similar conditions in females the sex chromatin was seen in from 66 to 82 per cent of nuclei, with an average incidence of 75 per cent. The average incidence of the sex chromatin in 18 normal tissues of female subjects was 74 per cent (Moore and Barr, 1954). Differences in the technical quality of the sections were probably responsible for most of the variation from one specimen to another.

The sex chromatin usually took the form of a plano-convex mass, adherent to the inner surface of the nuclear membrane. Nuclei in a cutaneous papilloma, a uterine leiomyoma, a thyroid adenoma and in a nerve cell of an ovarian dermoid cyst are illustrated in Fig. 1, 3, 4 and 5. The sex chromatin of smooth muscle

TABLE I.—*Incidence (per cent) of Sex Chromatin.*

| Histogenic classification. | Pathological diagnosis. | ♀. | ♂. |
|---|---|---|---|
| Glandular epithelium | Gastric polypus | 78 (2) | — |
| | Multiple polyposis of colon | 78 | 11 (2) |
| | Rectal polypus | 68 | 6 (6) |
| | Papilloma of urinary bladder | — | 6 |
| | Hypertrophy of prostate | — | 6 (3) |
| | Endocervical polyp | 66 | — |
| | Fibroadenoma of breast | 71 (2) | — |
| | Duct papilloma of breast | 74 | — |
| | Cystic lobular hyperplasia of breast | 72 | — |
| | Mazoplasia of breast | 70 | 4 (2) |
| | Adenoma of sweat gland | 75 (2) | 9 |
| | Sebaceous cyst | — | 5 (3) |
| | Arrhenoblastoma | 80 | — |
| | Leydig cell tumour | — | 4 |
| | Chromophobe adenoma of pituitary | 76 | — |
| | Colloid adenoma of thyroid | 69 | 4 |
| | Foetal adenoma of thyroid | 76 (2) | — |
| | Adenomatous goitre | 77 (3) | 15 |
| | Parathyroid adenoma | — | 18 |
| Non-glandular epithelium | Cutaneous papilloma | 75 (7) | 6 (12) |
| | Senile keratosis | 69 (2) | 6 |
| | Molluscum contagiosum | — | 6 |
| | Leukoplakia of buccal mucosa | 73 (2) | 4 (3) |
| | Papilloma of tonsillar fossa | — | 9 |
| | Condyloma acuminatum | 73 (2) | — |
| Melanin-forming tissue | Intradermal naevus | 79 (5) | 7 (7) |
| | Compound naevus | 75 (2) | — |
| | Blue naevus | 77 | — |
| | Junctional naevus | 69 | — |
| Vascular tissue | Capillary haemangioma | 76 | 7 |
| | Cavernous haemangioma | — | 3 |
| Muscle | Leiomyoma of uterus | 79 (7) | — |
| | Leiomyoma of cervix | 75 | — |
| | Leiomyoma of broad ligament | 79 | — |
| | Leiomyoma of stomach | 75 | 4 (3) |
| Connective tissue | Keloid | 76 | — |
| | Dermatofibroma | 68 | 4 (3) |
| | Pedunculated fibroma of hard palate | 71 | — |
| | Chondroma | — | 3 |
| | Osteochondroma | 67 | 4 |
| Embryonal and mixed tissues | Dermoid cyst of forehead | — | 2 |
| | Branchial cleft cyst | 71 | 4 |
| | Mixed tumour of parotid | 75 (3) | 8 (2) |
| | Dermoid cyst of ovary | 74 (4) | — |

tumours was not located at the pole of the nucleus as often as it is in normal smooth muscle cells. With this minor difference, its position in the nucleus was similar in normal tissues and in the tumours.

Measurements of the female sex chromatin in normal cells of various tissues are recorded in Table II. The mean size, considering the tissues together, was $0{\cdot}7 \times 1{\cdot}2\ \mu$. The size of the female sex chromatin appears to be similar in monkey and man since Prince, Graham and Barr (1955) found the mean size of the sex chromatin to be $0{\cdot}8 \times 1{\cdot}1\ \mu$ in various tissues of *Macacus rhesus*. Some of the size variation, as recorded in Table II, is undoubtedly caused by sampling and by errors in measuring such a small object. Nevertheless, we suspect that the female sex chromatin is a little larger in some tissues than in others since it was found to be relatively large in the adrenal cortex, for example, in man, monkey and in the cat (Graham and Barr, 1952).

TABLE II.—*Size of the Sex Chromatin in Normal Tissues of Females.*

| Tissue. | Mean size of sex chromatin ($\mu$). |
|---|---|
| Epidermis | 0·7 × 1·2 |
| Umbilical cord (surface epithelium) | 0·8 × 1·1 |
| Surface epithelium, gastric mucosa | 0·7 × 1·3 |
| Surface epithelium, duodenal mucosa | 0·6 × 1·1 |
| Parenchymal cells of liver | 0·7 × 1·3 |
| Acinar and duct epithelium, pancreas | 0·7 × 1·2 |
| Convoluted tubules of kidney | 0·6 × 1·2 |
| Epithelium, urinary bladder | 0·9 × 1·1 |
| Stromal cells of ovary | 0·8 × 1·2 |
| Glandular epithelium of uterus | 0·7 × 1·1 |
| Large lymphocytes of splenic nodules | 0·6 × 1·1 |
| Cartilage cells | 0·8 × 1·4 |
| Skeletal muscle | 0·7 × 1·3 |
| Cardiac muscle | 0·6 × 1·2 |
| Smooth muscle of uterus | 0·7 × 1·1 |
| Pars distalis of pituitary | 0·8 × 1·3 |
| Adrenal cortex | 0·9 × 1·3 |
| Adrenal medulla | 0·7 × 1·1 |
| Thyroid epithelium | 0·7 × 1·4 |
| Islets of Langerhans | 0·7 × 1·3 |
| Average | 0·7 × 1·2 |

Measurements of the female sex chromatin in cells of benign tumours and related conditions are shown in Table III. Considering the various abnormal tissues together, the average size of the sex chromatin was 0·8 × 1·2 $\mu$. Such measurements on a considerable number of benign tumours and their tissues of origin would be necessary to learn whether the size of the sex chromatin is altered significantly in benign tumours. From the work done thus far it may be inferred that any possible difference must be very small and difficult to demonstrate.

TABLE III.—*Size of the Sex Chromatin in Benign Tumours and Related Conditions in Females.*

| Tissue. | Mean size of sex chromatin ($\mu$). |
|---|---|
| Cutaneous papilloma | 0·8 × 1·2 |
| Intradermal naevus | 0·6 × 1·1 |
| Junctional naevus | 0·8 × 1·1 |
| Senile keratosis | 0·9 × 1·2 |
| Keloid | 0·9 × 1·1 |
| Leukoplakia of buccal mucosa | 0·8 × 1·2 |
| Gastric polypus | 0·8 × 1·3 |
| Multiple polyposis of colon | 0·8 × 1·2 |
| Osteochondroma | 0·8 × 1·4 |
| Leiomyoma of uterus | 0·7 × 1·3 |
| Dermoid cyst of ovary | 0·8 × 1·2 |
| Mixed tumour of parotid | 0·8 × 1·3 |
| Chromophobe adenoma of pituitary | 0·7 × 1·2 |
| Adenomatous goitre | 0·8 × 1·3 |
| Foetal adenoma of thyroid | 0·7 × 1·3 |
| Average | 0·8 × 1·2 |

(b) *Nuclei of benign tumours and related conditions in males.*

With one exception, noted below, the specimens obtained from male subjects showed a typical male nuclear structure (Fig. 2). A mass of chromatin that was rather larger than the other chromatin particles was seen in an average of 6 per cent of nuclei, varying in individual specimens from 2 to 18 per cent. One cannot be certain that these masses of chromatin represent the male sex chromatin derived from the XY-chromosomes. Although the figures for the incidence of sex chromatin in specimens from males are, on this account, only approximations they serve to illustrate the definite difference in nuclear structure between specimens obtained from females and those obtained from males. The figures are similar to those that we have recorded previously for normal male tissues, where a chromatin mass that might represent the sex chromatin of male nuclei was seen in an average of 8 per cent of nuclei, with the incidence varying in individual specimens from 1 to 21 per cent. In both normal tissues and benign lesions there is a wide range, between a high figure of about 20 per cent for males and a low figure of about 60 per cent for females, separating the incidence of sex chromatin in the two sexes.

A teratoma of the testis was studied but was omitted from Table I because it does not fall under the heading of benign tumours. However, the observations on this tumour are sufficiently significant to justify a brief reference to it at this time. This teratoma developed in the right testis of a 23-year-old and was removed at operation. It contained both epithelial and mesodermal elements. Much of the tumour was unsuitable for detailed cytological study. Certain areas were good for cytological detail, however, and in these favourable locations the nuclei had a typical female structure with a mass of sex chromatin occurring in about 70 per cent of the nuclei. The nucleus of a fibroblast-type cell in the teratoma, with typical female sex chromatin, is illustrated in Fig. 6.

### DISCUSSION.

The nuclei of benign neoplasms are generally thought to be similar to those of normal tissues. They have been described as larger, in some instances, than nuclei of the normal tissue in which the neoplasm arose (Ehrich, 1936). However, the mitotic and chromosomal aberrations that may occur in malignant tumours have not been described as a feature of benign neoplasms. To this may now be added the further item that the nuclei of benign tumours and normal tissues in man are similar with respect to their morphological characteristic of sex.

The finding of typical female nuclei in an arrhenoblastoma of the ovary (Table I), recalls a histogenetic hypothesis which was advanced by Schiller (1940) for these tumours. He suggested that a cell may lose an X-chromosome during a mitotic division, thus acquiring the potentiality of producing a tissue along male

---

### EXPLANATION OF PLATE.

(The magnification of all photomicrographs is × 2000. The sex chromatin is indicated by an arrow.)

FIG. 1.—Cutaneous papilloma, female. Feulgen stain.
FIG. 2.—Cutaneous papilloma, male. Feulgen stain.
FIG. 3.—Leiomyoma of uterus. Haematoxylin and eosin stain.
FIG. 4.—Thyroid adenoma, female. Feulgen stain.
FIG. 5.—Nerve cell in dermoid cyst of ovary. Cresyl violet stain.
FIG. 6.—Fibroblast in a teratoma of the testis. Feulgen stain.

1
2
3
4
5
6

structural lines. The arrhenoblastoma in this series was submitted for study by Dr. A. R. Sohval, of New York City. It was a trabeculated type of arrhenoblastoma containing pseudotubular structures, and of excellent technical quality The interstitial cells had a typical female structure. It is inferred that they bear the XX-sex chromosome complex and that the hypothesis of Schiller does not apply to this particular specimen. A more detailed description of this tumour, together with an account of nuclear structure in a number of pathological conditions, will be published shortly (Sohval and Gaines, 1955).

The finding of female nuclei in a teratoma of the testis raises questions concerning the histogenesis of these tumours that cannot be answered at this time. Hunter and Lennox (1954) found that of 12 teratomata in women all had female-type nuclei, while of 9 teratomata in males 5 had female-type nuclei and 4 had male-type nuclei. Cruickshank (1955) studied squamous-cell formations in 5 mediastinal teratomata. Two of the hosts were female and the tumour nuclei were also female. Three of the hosts were male, and the tumour nuclei were male in two instances and female in the other. Because of these observations the view, currently discounted, that a teratoma may be a unique tumour arising from the fusion of gametes or other haploid cells merits renewed consideration.

### SUMMARY.

Nuclei of benign tumours and related conditions in man were studied with respect to their sex characteristics. Sixty-five specimens were from female subjects and all showed typical female-type nuclei. Sixty-one specimens from males contained typical male-type nuclei. It is concluded that the sex characteristics of nuclei, i.e. the presence of a well-developed mass of sex chromatin in females only, is similar in benign tumours and related conditions and in normal tissues.

The finding of typical female-type nuclei in an arrhenoblastoma of the ovary discounts the hypothesis that these tumours are generally derived from a cell which has lost an X-chromosome. On the other hand, the presence of female-type nuclei in a teratoma of the testis re-opens the problem of the histogenesis of this type of tumour.

This work was supported by a grant from the National Cancer Institute of Canada. We are grateful to the following pathologists for placing specimens at our disposal and for allowing us to make use of their pathological diagnoses: Professor J. H. Fisher, Department of Pathology, University of Western Ontario; Professor J. C. Paterson, Department of Medical Research, University of Western Ontario; and Dr. W. M. Wilson, Regional Laboratory, Ontario Department of Health, London, Ontario.

### REFERENCES.

BARR, M. L.—(1954) *Surg. Gynec. Obstet.*, **99**, 184.
CASPERSSON, T.—(1950) 'Cell Growth and Cell Function.' New York (W. W. Norton & Co., Inc.).
*Idem* AND SANTESSON, L.—(1942) *Acta radiol., Stockh.*, Suppl. 46, 1.
CRUICKSHANK, D. B.—(1955) *Lancet*, i, 253.
EHRICH, W. E.—(1936) *Amer. J. med. Sci.*, **192**, 772.
EMERY, J. L. AND MCMILLAN, M.—(1954) *J. Path. Bact.*, **68**, 17.

Gatenby, J. B. and Beams, H. W.—(1950) 'The Microtomist's Vade-Mecum (Bolles Lee).' London (J. & A. Churchill, Ltd.).
Graham, M. A. and Barr, M. L.—(1952) *Anat. Rec.*, **112**, 709,
Gurr, E.—(1953) 'A Practical Manual of Medical and Biological Staining Techniques.' New York (Interscience Publishers, Inc.).
Hunter, W. F. and Lennox, B.—(1954) *Lancet*, ii, 633.
*Iidem* and Pearson, M. G.—(1954) *Ibid.*, i, 372.
Marberger, E. and Nelson, W. O.—(1954) *Anat. Rec.*, **118**, 399.
Moore, K. L.—(1955) *Ibid.*, **121**, 409.
*Idem* and Barr, M. L.—(1954) *Acta anat.*, **21**, 197.
*Idem.*, Graham, M. A. and Barr, M. L.—(1953) *Surg. Gynec. Obstet.*, **96**, 641.
Polani, P. E., Hunter, W. F. and Lennox, B.—(1954) *Lancet*, ii, 120.
Prince, R. H., Graham, M. A. and Barr, M. L.—(1955) *Anat. Rec.* (in press).
Schiller, W.—(1940) *New int. Clin.*, **3**, 86.
Sohval, A. R. and Gaines, J. A.—(1955)*Cancer* (in press).
*Iidem* and Gabrilove, J. L.—(1955) *Amer. J. Obstet. Gynec.* (in press).
Wilkins, L., Grumbach, M. M. and Van Wyk, J. J.—(1954) *J. clin. Endocrin.* **14**, 1270.